# Evidence-Based Higher Education – Is the Learning Styles ‘Myth’ Important?

**DOI:** 10.3389/fpsyg.2017.00444

**Published:** 2017-03-27

**Authors:** Philip M. Newton, Mahallad Miah

**Affiliations:** Swansea University Medical SchoolSwansea, UK

**Keywords:** Learning Styles, evidence-based education, VARK, Kolb, neuromyths

## Abstract

The basic idea behind the use of ‘Learning Styles’ is that learners can be categorized into one or more ‘styles’ (e.g., Visual, Auditory, Converger) and that teaching students according to their style will result in improved learning. This idea has been repeatedly tested and there is currently no evidence to support it. Despite this, belief in the use of Learning Styles appears to be widespread amongst schoolteachers and persists in the research literature. This mismatch between evidence and practice has provoked controversy, and some have labeled Learning Styles a ‘myth.’ In this study, we used a survey of academics in UK Higher Education (*n* = 114) to try and go beyond the controversy by quantifying belief and, crucially, actual use of Learning Styles. We also attempted to understand how academics view the potential harms associated with the use of Learning Styles. We found that general belief in the use of Learning Styles was high (58%), but lower than in similar previous studies, continuing an overall downward trend in recent years. Critically the percentage of respondents who reported actually using Learning Styles (33%) was much lower than those who reported believing in their use. Far more reported using a number of techniques that are demonstrably evidence-based. Academics agreed with all the posited weaknesses and harms of Learning Styles theory, agreeing most strongly that the basic theory of Learning Styles is conceptually flawed. However, a substantial number of participants (32%) stated that they would continue to use Learning Styles despite being presented with the lack of an evidence base to support them, suggesting that ‘debunking’ Learning Styles may not be effective. We argue that the interests of all may be better served by promoting evidence-based approaches to Higher Education.

## Introduction

The use of so-called ‘Learning Styles’ in education has caused controversy. The basis for the use of Learning Styles is that individual difference between learners can supposedly be captured by diagnostic instruments which classify learners into ‘styles’ such as ‘visual,’ ‘kinaesthetic,’ ‘assimilator,’ etc. According to many, but not all, interpretations of Learning Styles theory, to teach individuals using methods which are matched to their ‘Learning Style’ will result in improved learning (Pashler et al., 2008). This interpretation is fairly straightforward to test, and, although there are over 70 different instruments for classifying Learning Styles (Coffield et al., 2004) the current status of the literature is that there is no evidence to support the use of Learning Styles in this way (Pashler et al., 2008; Rohrer and Pashler, 2012). This has lead to Learning Styles being widely classified as a ‘myth’ (Geake, 2008; Riener and Willingham, 2010; Lilienfeld et al., 2011; Dekker et al., 2012; Pasquinelli, 2012; Rato et al., 2013; Howard-Jones, 2014).

Despite this lack of evidence, it appears that belief in the use of Learning Styles is common amongst schoolteachers – A 2012 study demonstrated that 93% of schoolteachers in the UK agree with the statement “Individuals learn better when they receive information in their preferred Learning Style (e.g., auditory, visual, kinaesthetic) (Dekker et al., 2012).” A 2014 survey reported that 76% of UK schoolteachers ‘used Learning Styles’ and most stated that to do so benefited their pupils in some way (Simmonds, 2014). A study of Higher Education faculty in the USA showed that 64% agreed with the statement “Does teaching to a student’s learning style enhance learning?” (Dandy and Bendersky, 2014). A recent study demonstrated that current research papers ‘about’ Learning Styles, in the higher education research literature, overwhelmingly endorsed their use despite the lack of evidence described above (Newton, 2015). Most of this endorsement was implicit and most of the research did not actually test Learning Styles, rather proceeded on the assumption that their use was a ‘good thing.’ For example, researchers would ask a group of students to complete a Learning Styles questionnaire, and then make recommendations for curriculum reform based upon the results.

This mismatch between the empirical evidence and belief in Learning Styles, alongside the persistence of Learning Styles in the wider literature, has lead to tension and controversy. There have been numerous publications in the mainstream media attempting to explain the limitations of Learning Styles (e.g., Singal, 2015; Goldhill, 2016) and rebuttals from practitioners who believe that the theory of Learning Styles continues to offer something useful and/or that criticism of them is invalid (e.g., Black, 2016). Some of the original proponents of the concept have self-published their own defense of Learning Styles, e.g., (Felder, 2010; Fleming, 2012).

The continued use of Learning Styles is, in theory, associated with a number of harms (Pashler et al., 2008; Riener and Willingham, 2010; Dekker et al., 2012; Rohrer and Pashler, 2012; Dandy and Bendersky, 2014; Willingham et al., 2015). These include a ‘pigeonholing’ of learners according to invalid criteria, for example a ‘visual learner’ may be dissuaded from pursuing subjects which do not appear to match their diagnosed Learning Style (e.g., learning music), and/or may become overconfident in their ability to master subjects perceived as matching their Learning Style. Other proposed harms include wasting resources on an ineffective method, undermining the credibility of education research/practice and the creation of unrealistic expectations of teachers by students.

This study aimed at asking first whether academics in UK Higher Education also believe in Learning Styles. We then attempted to go beyond the controversy and ask whether academics actually use Learning Styles, and how seriously they rate the proposed harms associated with the use of Learning Styles, with the aim of understanding how best to address the persistence of Learning Styles in education. In addition, we compared belief in/use of Learning Styles to some educational techniques whose use is supported by good research evidence, to put the use of, and belief in, Learning Styles into context.

We found that belief in the use of Learning Styles was high (58% of participants), but that actual use of Learning Styles was much lower (33%) and lower than other techniques which are demonstrably effective. The most compelling weakness/harm associated with Learning Styles was a simple theoretical weakness; 90% of participants agreed that Learning Styles are conceptually flawed.

## Materials and Methods

Data were collected using an online questionnaire distributed to Higher Education institutions in the UK. Ethical approval for the study was given by the local Research Ethics Committee at Swansea University with informed consent from all subjects.

### Participants

The survey was distributed via email. Distribution was undertaken indirectly; emails were sent to individuals at eight different Higher Education institutions across the UK. Those persons were known to the corresponding author as colleagues in Higher Education but not through work related to Learning Styles. Those individuals were asked to send the survey on to internal email distribution lists of academics involved in Higher Education using the following invitation text (approved by the ethics committee) “You are invited to participate in a short anonymous survey about teaching methods in Higher Education. It will take approximately 10–15 min to complete. It is aimed at academics in Higher Education,” followed by a link to the survey which was entitled “Teaching Methods in Higher Education.” Thus the survey was not directly distributed by the authors and did not contain the phrase ‘Learning Styles’ anywhere in the title or introductory text. These strategies of indirect distribution, voluntary completion and deliberately not using the term ‘Learning Styles’ in the title were based upon similar strategies used in similar studies (Dekker et al., 2012; Dandy and Bendersky, 2014) and were aimed at avoiding biasing and/or polarizing the participant pool, given the aforementioned controversy associated with the literature on Learning Styles. Although this inevitably results in a convenience sample (we do not know how many people the survey as sent to or how many responded), this was preferable to distributing a survey that was expressly about Learning Styles (which may have put off those who are already familiar with the concept). The survey remained open for 2 months (which included the end-of-year holiday period) and was closed once we had over 100 participants who had fully completed the survey, to ensure a sample size equivalent to similar studies (Dekker et al., 2012; Dandy and Bendersky, 2014).

One hundred sixty-one participants started the survey, with 114 completing the survey up to the final (optional) question about demographics. This meant that 29% of participants did not complete, which is slightly better than the average dropout rate of 30% for online surveys (Galesic, 2006). Question-by-question analysis revealed that the majority of these non-completers (79%) did not progress beyond the very first ranking question (ranking the effectiveness of teaching methods) and thus did not complete the majority of the survey, including answering those questions about Learning Styles. Participants had been teaching in Higher Education for an average of 11 years (*SD* = 9.8). Participants were asked to self-report their academic discipline. Simple coding of these revealed that participants came from a wide variety of disciplines, including Life and Physical Sciences (26%), Arts, humanities and languages (24%), Healthcare professions (medicine, nursing, pharmacy, etc.) (16%), Social Sciences (10%), Business and Law (5%).

### Materials and Procedure

The lack of an evidence base for Learning Styles has been described numerous times in the literature, and these papers have suggested that there may be harms associated with the use of Learning Styles (Pashler et al., 2008; Riener and Willingham, 2010; Dekker et al., 2012; Rohrer and Pashler, 2012; Dandy and Bendersky, 2014; Willingham et al., 2015). We reviewed these publications to identify commonly posited harms. We then constructed a questionnaire using LimeSurvey^TM^. All the survey questions are available via the Supplementary Material. Key aspects of the structure and design are described below. The survey was piloted by five academics from Medical and Life Sciences, all of whom were aware of the lack of evidence regarding Learning Styles. They were asked to comment on general clarity and were specifically asked to comment on the section regarding the evidence for the use of Learning Styles and whether it would disengage participants (see below). Key concepts in the survey were addressed twice, from different approaches, so as to ensure the quality of data obtained.

Participants were first asked to confirm that they were academics in Higher Education. They were then asked about their use of five teaching methods, four of which are supported by research evidence [Worked Examples, Feedback, Microteaching and Peer Teaching (Hattie, 2009)] and Learning Styles. They were then asked to rank these methods by efficacy.

We then asked participants about their use of Learning Styles, both generally and the use of specific classifications (VARK, Kolb, Felder, Honey and Mumford). For each of these individual Learning Styles classifications we identified, in our question, the individual styles that result (e.g., active/reflective, etc., from Felder). Thus participants were fully oriented to what was meant by ‘Learning Styles’ before we went on to ask them about the efficacy of Learning Styles. To allow comparisons with existing literature, we used the same question as Dekker et al. (2012) “Rate your agreement with this statement ‘Individuals learn better when they receive information in their preferred Learning Style (e.g., auditory, visual, kinaesthetic).”’

We then explained to participants about the lack of an evidence base for the use of Learning Styles, including the work of Coffield et al. (2004), Pashler et al. (2008), Rohrer and Pashler (2012), Willingham et al. (2015). We explained the difference between learning preferences and Learning Style, and made it clear that there was specifically no evidence to support the ‘matching’ of teaching methods to individual Learning Styles. We explained that this fact may be surprising, and that participants would be free to enter any comments they had at the end of the survey. Those academics who piloted the initial survey were specifically asked to comment on this aspect of the survey to ensure that it was neutral and objective.

We then asked participants to rate their agreement with some of the proposed harms associated with the use of Learning Styles. Mixed into the questions about harms were some proposed reasons to use Learning Styles, regardless of the evidence. These questions were interspersed so as to avoid ‘acquiescence bias’ (Sax et al., 2003). Agreement was measured on a 5-point Likert scale.

Finally, participants were asked for some basic demographic information and then offered the opportunity to provide free-text comments on the content of the survey.

Quantitative data were analyzed by non-parametric methods; specific tests are described in the results. Percentages of participants agreeing, or disagreeing, with a particular statement were calculated by collapsing the two relevant statements within the Likert scale (e.g., ‘Strongly Agree and Agree’ were collapsed into a single value). Qualitative data (free-text comments) were analyzed using a simple ground-up thematic analysis (Braun and Clarke, 2006) to identify common themes. Both authors independently read and re-read the comments to identify their own common themes. The authors then met and discussed these, arriving at agreed common themes and quantifying the numbers of participants who had raised comments for each theme. Many participant comments were pertinent to more than one theme.

## Results

### Belief vs. Use; Do Teachers in Higher Education Actually Use Learning Styles?

**FIGURE 1:**
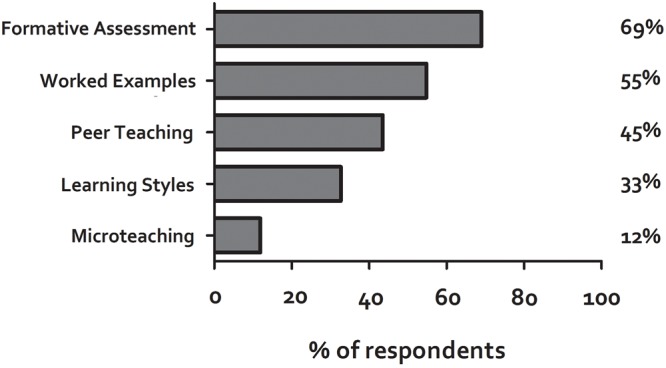
**Use of various teaching methods in the last 12 months.** Academics were asked which of the methods they had used in the last 12 months. Four of the methods were accompanied by a brief description: Formative Assessment (practice tests), Peer Teaching (students teaching each other), Learning Styles (matching teaching to student Learning Styles). Microteaching (peer review by educators using recorded teaching).

We addressed this question from two perspectives. Academics were asked to identify which teaching methods, from a list of 5, they had used in the last 12 months. Results are shown in **Figure 1**. Thirty-three percent of participants reported having used Learning Styles in the last 12 months, but this was lower than the evidence-based techniques of formative assessment, worked examples, and peer teaching. Participants were then asked “have you ever administered a Learning Styles questionnaire to your students” and were given four specific examples along with the ‘styles’ identified by those examples. The examples chosen were those most commonly found in a recent study of the literature on Learning Styles (Newton, 2015). Participants were also given the option to check ‘other’ and identify any other types of Learning Styles questionnaire that they might have used. 33.1% of participants had given their students any sort of Learning Styles Questionnaire, with the response for individual classifications being 18.5% (Honey and Mumford), 14.5% (Kolb), 12.9% (VARK), and 1.6% (Felder).

**FIGURE 2:**
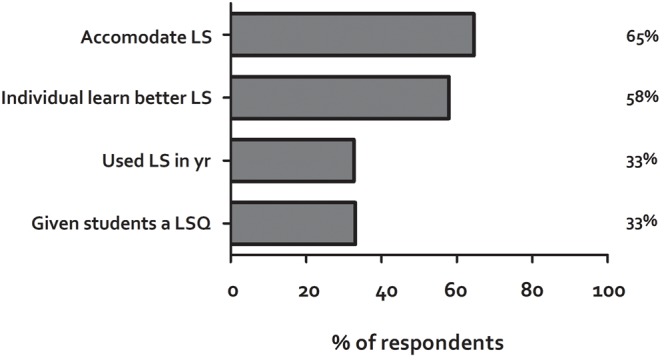
**Belief in use of Learning Styles.** At different points throughout the survey, participants were asked to rate their agreement with the statements regarding their belief in, and their actual use of, Learning Styles. These questions were asked prior to informing participants about the lack of evidence for the use of Learning Styles. When asked if they believed in the use of Learning Styles^1,2^, approximately two thirds of participants agreed, whereas when asked specifically about actual use^3,4^, agreement dropped to one-third. ^1^*Rate your agreement with this statement: Individuals learn better when they receive information in their preferred Learning Style (Individuals learn better LS)*. ^2^*Rate your agreement with the statement: I try to organize my teaching to accommodate different Learning Styles (Accomodate LS)*. ^3^*Have you ever administered a Learning Styles questionnaire to your students? If so, please state which one (Given students a LSQ)*. ^4^*Which of these teaching methods have you used in the last 12 months? (Used LS in year)*.

We subsequently asked two, more general, questions about Learning Styles. The first of these was the same as that used by Dekker et al. “Individuals learn better when they receive information in their preferred Learning Style (e.g., auditory, visual, kinaesthetic),” with which 58% agreed. The second was “I try to organize my teaching to accommodate different student Learning Styles (e.g., visual, kinaesthetic, assimilator/converger),” with which 64% of participants agreed. These data show a contrast between a general belief in the use of Learning Styles, which is much higher than actual use (**Figure 2**).

### Possible Harms Associated with the Use of Learning Styles

**FIGURE 3:**
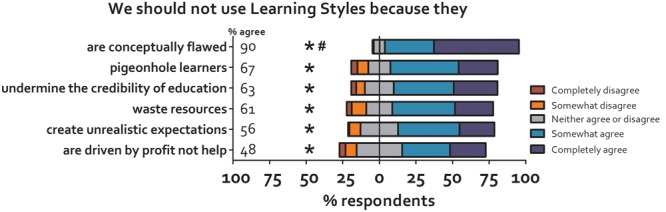
**Participants were asked to rate their agreement with various difficulties that have been proposed to result from the use of Learning Styles.** Participants agreed with all the proposed harms but there was a stronger agreement (compared to other options) with the idea that the use of Learning Styles is conceptually flawed. ^∗^, significantly different from median of ‘3’ (1-sample Wilcoxon Signed Rank test). #, different from other statements (Kruskal–Wallis test).

There was significant agreement with all the proposed difficulties associated with the use of Learning Styles, as shown in **Figure 3**. However, compared to the other proposed harms, participants showed stronger agreement with the statement “The theory of Learning Styles is conceptually flawed” – it does not account for the complexity of ‘understanding.’ It is not possible to teach complex concepts such as mathematics or languages by presenting them in only one style. In addition, some information cannot be presented in a single style (e.g., teaching medical students to recognize heart sounds would be impossible using visual methods, whereas teaching them to recognize different skin rashes would be impossible using sounds). In this section of the survey we also included two questions that were not about proposed harms. Forty-six percent of participants agreed with the statement “Even though there is no ‘evidence base’ to support the use of Learning Styles, it is my experience that their use in my teaching benefits student learning,” while 70% agreed that “In my experience, students believe, rightly or wrongly, that they have a particular Learning Style.”

### Ranking of Proposed Harms

Having asked participants to rate their agreement (or not) with the various harms associated with the use of Learning Styles, we then asked participants to “Rank the aforementioned factors in terms of how compelling they are as reasons not to use Learning Styles” (1, most compelling, 6, least compelling) and to “only rank those factors which you agree with.” There is not universal agreement on the analysis of ranking data and so we analyzed these data in two simple, descriptive ways. The first was to determine how frequently each harm appeared as the top ranked reason. The second was to calculate a ranking score, such that the top ranked harm was scored 6, and the lowest ranked scored 1, and then to sum these across the participants. Both are shown in **Table 1**. Results from both methods were similar and agreed with the prior analysis (**Figure 3**), with participants most concerned about the basic conceptual flaws associated with the use of Learning Styles, alongside a potential pigeonholing of learners into a particular style.

**Table 1:** Ranking of proposed harms as compelling reasons not to use Learning Styles.

	Ranking score	# Times top ranked
Waste resources that could be used elsewhere	302 (4)	11 (4)
Pigeonhole learners	445 (2)	34 (1)
Understanding more complex than Learning Styles	455 (1)	33 (2)
Profit motive of those selling Learning Styles instruments	191 (6)	7 (5=)
Unrealistic expectations of teachers	366 (3)	14 (3)
Credibility of education as a discipline	257 (5)	7 (5=)

*Ranking score was calculated individually whereby the top ranked harm was scored 6, and the lowest ranked scored 1. These were then totaled across the participants. Figures in parentheses indicate the top (1) to bottom (6) ranked by each method.*

### Continued Use of Learning Styles?

Toward the end of the questionnaire, we asked participants two question to determine whether the completion of the questionnaire had made any difference to their understanding of the evidence base for the use of Learning Styles. Participants were first asked to rate their agreement with the statement “Completing this questionnaire has helped me understand the lack of any evidence base to support the use of Learning Styles.” The 64% agreed while 9% disagreed and 27% neither agreed or disagreed.

**FIGURE 4:**
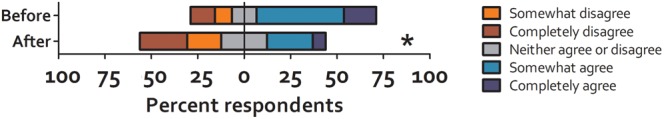
**The completion of the survey instrument associated with a change of participants views of Learning Styles.** At the beginning of the study, participants were asked to rate their agreement with the statement “I try to organize my teaching to accommodate different student Learning Styles (e.g., visual, kinaesthetic, assimilator/converger),” and 64% agreed. At the end of the study, participants were asked “In light of the information presented, rate your agreement with the following statement – ‘I plan to try and account for individual student Learning Styles in my teaching,”’ and 32% agreed. ^∗^, a Wilcoxon signed rank test revealed a statistically significant difference in the pattern of response (*P* < 0.0001, *W* = -1977).

Participants were then asked “In light of the information presented, rate your agreement with the following statement – ‘I plan to try and account for individual student Learning Styles in my teaching.”’ 31.6% agreed, 43.9% disagreed, and 23.6% neither agreed or disagreed. The results from this question were compared to those obtained before the evidence was presented, when participants were asked to rate their level of agreement with this statement “I try to organize my teaching to accommodate different student Learning Styles (e.g., visual, kinaesthetic, assimilator/converger).” The results, shown in **Figure 4**, show a statistically significant difference in the two sets of responses suggesting that completion of the questionnaire improved participants understanding of the lack of an evidence base for the use of Learning Styles and thus they were unlikely to continue using them. However, almost one-third of participants still agreed with the statement; they intended to continue using Learning Styles.

This then raised a series of interesting questions about why participants would persist in using Learning Styles despite having been presented with all the evidence showing that they are not effective (although participants were not specifically asked whether they would persist in the matching of instructional design to student Learning Style). The sample size here, although equivalent to previous studies, is modest and obviously the 32% are only a portion of that. Thus we were reluctant to undertake extensive *post hoc* analysis to identify relationships within the sample. However, in response to a reviewer’s suggestion we undertook a simple descriptive analysis of the profile of the 31.6% of participants who indicated that they would continue to account for Learning Styles and compare them to the 43.9% who said that they would not. When splitting the data into these two groups, we observed that almost all (94.4%) of those who said they would still use Learning Styles at the end of the survey had originally agreed with the question “I try to organize my teaching to accommodate different student Learning Styles (e.g., visual, kinaesthetic, assimilator/converger),” and no participants from that group had disagreed. In contrast, agreement was only 40% for the group that eventually said they would not use Learning Styles, while disagreement was 46%. A similar split was found for the question “Even though there is no ‘evidence base’ to support the use of Learning Styles, it is my experience that their use in my teaching benefits student learning”; for the group that would go on to say that they will still use Learning Styles, 89% agreed, while agreement was only 18% from the group that would go on to say they will not continue to use Learning Styles.

### Educational Research Literature

Finally we asked participants to rate their agreement with the statement “my educational practice is informed by the education research literature.” Forty-eight percent of participants agreed with the statement. A Spearman Rank Correlation test revealed no correlation between responses on that question and on the ‘Dekker’ question “Individuals learn better when they receive information in their preferred Learning Style (e.g., auditory, visual, kinaesthetic)” *r* = 0.07508, *P* = 0.4.

### Qualitative Comments

Forty-eight participants left free-text comments. The dominant common theme, raised by 23 participants was the need to use a variety of teaching methods in order to (for example) keep students engaged or to promote reflection. This theme was often stated in the context of ‘despite the evidence again showing a lack of effectiveness of Learning Styles.’ A related theme (13 participants) was that participants had a looser interpretation of ‘Learning Styles,’ for example that they referred simply to ‘styles of learning,’ while a second related theme from nine participants was they would still, despite the evidence, use Learning Styles and/or found them useful. Eight participants commented that they were aware of the lack of evidence base for the use of Learning Styles and eight participants also gave their own examples of why Learning Styles were conceptually flawed. Despite the careful piloting described above, a small number of participants (four) commented that the survey was biased against Learning Styles, while eight participants perceived some of the questions to be ‘leading.’ No specific ‘leading’ questions were identified but there was a substantial overlap between these two themes, with three of the comments about the survey being ‘biased against Learning Styles’ coming alongside, or as part of, a comment about questions being ‘leading,’ with an implied relationship between the two. An additional theme, from five participants, was thanks; for raising the issue and/or interesting content.

## Discussion

The first aim of this study was to determine how widespread belief in, and use of, Learning Styles is by academics in UK Higher Education. In a 2012 study, 93% of a sample of 137 UK school teachers agreed with the statement “Individuals learn better when they receive information in their preferred learning style (e.g., auditory, visual, kinesthetic).” In our sample of academics in UK Higher Education, 58% agreed with that same statement while 64% agreed with the similar, subsequent statement “I try to organize my teaching to accommodate different Learning Styles.” Thus a majority of academics in UK HE ‘believe’ in the use of Learning Styles although the figures are lower than in the 2012 study of schoolteachers. However, prior to asking these questions we asked some more direct questions about the actual use of Learning Styles instruments. Here the figures were much lower, with 33% of participants answering ‘yes’ to the statement “Have you ever administered a Learning Styles questionnaire to your students” and the same number stating that they had used ‘Learning Styles’ as a method in the last 12 months, where the method was defined as “matching teaching to individual student Learning Styles.” This value was lower than for a number of teaching methods that are evidence-based. Interestingly the most commonly used Learning Styles instrument was the Kolb Learning Styles Inventory; this is the Learning Styles classification that has been most frequently tested for evidence of such a ‘matching effect’ and where no evidence has been found (Pashler et al., 2008).

The empirical evidence is clear that there is currently no evidence to support the use of Learning Styles instruments in this way (Coffield et al., 2004; Pashler et al., 2008) and thus the fact that actual use of Learning Styles is lower than the use of demonstrably evidence-based methods could be considered reassuring, as could our finding that actual use is lower than ‘belief’ in the efficacy of Learning Styles. In addition, although we find that a majority of UK academics in Higher Education believe in the use of Learning Styles, the actual numbers observed are the lowest of any similar study. Studies examining belief in the use of Learning Styles have been carried out over the last few years in a number of different populations, and the overall trend is down, from 93% of UK schoolteachers in 2012 (Dekker), to 76% of UK schoolteachers in 2014 (Simmonds), 64% of HE academics in the US in 2014 (Dandy and Bendersky) to 58% here. There are obviously a number of caveats to consider before concluding that belief in the use of Learning Styles is declining; these studies have been conducted in different countries (US and UK), using teachers in different disciplines (school teachers and higher education). A follow-up, longitudinal study across different populations/contexts would be informative to address whether belief in the use of Learning Styles is truly declining, and to further understand whether actual use of Learning Styles is lower than ‘belief,’ as we have found here.

However, a more pessimistic interpretation of the data would be to focus on our finding that one-third of academics in UK higher education have, in the last year, used a method that was shown to be ineffective more than a decade earlier. The free-text comments give us some insight into the broader issue and perhaps a further hypothesis as to why the ‘myth’ of Learning Styles persists. The dominant theme was a stated need to use a diverse range of teaching methods. This is a separate issue to the use of Learning Styles and there was no suggestion in the survey that to *not* use Learning Styles was to advocate for all students to be taught the same way, and/or to use only one method of teaching. Neither of these approaches are advocated by the wider literature which seeks to ‘debunk’ Learning Styles, but it is clear from the abundance of comments on this theme that these two issues were related in the view of many of the participants. This is supported by the emergence of the related theme of ‘styles of learning rather than Learning Styles’; many participants had a looser definition of ‘Learning Styles’ than those introduced early in the survey. This finding leads us to urge caution and clarity in the continued ‘debunking’ of the ‘myth’ of Learning Styles. Learners obviously have preferences for how they learn. In addition, there is an obvious appeal to using a variety of teaching methods and in asking students to reflect on the ways in which they learn. However, these three concepts are unrelated to the (unsupported) idea that there is a benefit to learners from diagnosing their ‘Learning Style’ using one of the specific classifications (Coffield et al., 2004) and attempting to match teaching to those styles. However, these concepts were clearly linked in the mind of many of our participants.

Participants agreed with many of the statements describing proposed harms or weaknesses of Learning Styles. Part of our intention here was to understand which are the most compelling of these; all have, at least, a face validity if not empirical evidence to support them. As we attempt to ‘spread the word’ about Learning Styles and promote alternate, evidence-based approaches, it is useful to know where perceived weaknesses are with Learning Styles. Thus our aim was not so much to observe absolute rates of agreement with individual harms/weaknesses (we would expect to see agreement, given that participants had just been told of the lack of evidence for Learning Styles), but to identify any differences in rates of agreement between the individual statements. There was strongest agreement with the conceptual weaknesses associated with Learning Style theory; that it is not possible to teach ‘understanding’ using a particular style, or to capture certain types of learning in all styles. Weakest agreement was with the statement that “The continued promotion of Learning Styles as a product is exploiting students and their teachers, for the financial gain of those companies which sell access to, and training in, the various Learning Style questionnaires.” The difference between the ‘conceptual weakness’ and other weaknesses/harms was statistically significant, suggesting that, where efforts are being made to ‘debunk’ the ‘myth’ of Learning Styles, then an appeal to the simple conceptual problems may be the most compelling approach. This would also seem to fit with the data described above re: ‘belief vs. use’; although it is tempting to believe that individual students have a Learning Style than can be utilized to benefit their education, the conceptual flaws inherent in the theory mean that actually putting them into practice may prove challenging.

Completion of the questionnaire, which highlighted all of the problems associated with the use of Learning Styles, was clearly associated with a group-shift in the *stated* likelihood that the participant group would use Learning Styles, although we must also consider that, having been presented with all the evidence that Learning Styles are not effective, it seems reasonable to assume that some participants may succumb to some form of social desirability bias, wherein participants respond in the way that they perceive the researchers desire or expect (Nederhof, 1985). However, despite being presented with all the aforementioned evidence, approximately one-third of participants still agreed with the statement “In light of the information presented……‘I plan to try and account for individual student Learning Styles in my teaching.’” As described in the section “Introduction” there is an ongoing controversy, often played out via blogs and social media, about the use of Learning Styles, with some continuing to advocate for their use despite presentation of all the aforementioned evidence. It is even possible that to persist with a ‘myth debunking’ approach to Learning Styles may be counter-productive; the so-called ‘backfire effect’ describes a phenomenon wherein attempts to counter myths and misconceptions can result in a strengthening of belief in those myths. For example, 43% of the US population believe that the flu vaccine causes flu, and amongst that group are some who are very worried about the side effects of vaccines. Correcting the misconception that the vaccine causes flu is effective in reducing belief in the myth, yet reduces the likelihood that those who are concerned about vaccines will get vaccinated (Nyhan and Reifler, 2015). We observed that almost all those who said they would still use Learning Styles after completing the survey had originally said that they try to account for Learning Styles in their teaching. An interesting question for further study may be to ask, of those who are currently using Learning Styles, whether being presented with the (lack of) evidence regarding their use makes it *more* likely that those academics will continue to use them? In addition, it may be informative to use an in-depth qualitative approach that would allow us to understand, in detail, what it is about Learning Styles that continues to appeal.

Instead of focusing on Learning Styles, it may be more productive for all, most importantly for students, to focus on the use of teaching and development activities which are demonstrably effective. For example, the use of microteaching, a simple, multi-peer review activity, the effectiveness of which has been repeatedly demonstrated in teacher-training settings (Yeany and Padilla, 1986). Only 12% of survey participants here stated that they had used microteaching within the last 12 months, yet to do so would be relatively straightforward; it is little more than the application of a few more peers to an episode of peer-observation; something that is routinely undertaken by academics in UK Higher Education. This finding may be confounded by participants simply not being aware that ‘microteaching’ means, basically, ‘multi-peer observation and feedback,’ although this was explained twice in the survey itself.

Further support for an approach focused on raising awareness comes from our finding (**Figure 1**) that, as a group, participants stated use of different teaching methods mapped directly on to their perceived usefulness (e.g., the most commonly used technique was formative assessment which was also perceived as the most effective). It seems reasonable to infer a causative relationship between these two observations, i.e., that participants use techniques which they consider to be effective, and thus if we can raise awareness of techniques which are demonstrably effective, then their use will increase.

There are some limitations to our study. A review of factors associated with dropouts from online surveys (Galesic, 2006) observed that the average dropout rate amongst general-invitation online surveys (such as this one) is ∼30%, and so our dropout rate is entirely within expectations, although upon reflection we could perhaps have designed the instrument in a way that reduced dropout. A number of factors are associated with higher dropout rates, including the participant’s level of interest in the topic and the presence of ‘matrix questions.’ As described in the methods, we deliberately avoid entitling the survey as being about ‘Learning Styles’ to avoid biasing the responses, and a detailed analysis of the participation rate for each question revealed that the majority of dropouts occurred very early in the survey, after being asked to rank the effectiveness of the five teaching methods; a question potentially requiring higher effort than the others. An additional point reviewed by Galesic (2006) is the evidence that the quality of responses tails off for the items preceding the actual dropout point, thus the fact that participation rate remained steady after this early dropout is reassuring. It would also have been helpful to have a larger sample size. Although ours was equivalent to that in similar studies (Dekker et al., 2012; Dandy and Bendersky, 2014) we may have been able to tease out more detail from the responses with a larger sample size, for example to determine whether ‘belief’ in Learning Styles was associated with any of the demographics factors (e.g., subject discipline, or age) to get a deeper understanding of why and where Learning Styles persist.

In summary, we found that 58% of academics in UK Higher Education believe that Learning Styles are effective, but only about a third actually use them, a lower percentage than use other, demonstrably evidence-based techniques. Ninety percent of academics agreed that there is a basic conceptual flaw with Learning Styles Theory. These data suggest that, although there is an ongoing controversy about Learning Styles, their actual use may be low, and further attempts to educate colleagues about this limitation might best focus on the fundamental conceptual limitations of Learning Styles theory. However, approximately one-third of academics stated that they would continue to use Learning Styles despite being presented with all the evidence. Thus it may be better still to focus on the promotion of techniques that are demonstrably effective.

## Author Contributions

PN conceived the study, PN and MM designed the questionnaire, PN piloted and distributed the questionnaire, PN and MM analyzed the data, PN wrote the manuscript.

## Conflict of Interest Statement

The authors declare that the research was conducted in the absence of any commercial or financial relationships that could be construed as a potential conflict of interest.
