# Supplementary material for: Evidence-Based Higher Education – Is the Learning Styles ‘Myth’ Important?
Source: Front Psychol. 2017 Mar 27;8:444. doi: 10.3389/fpsyg.2017.00444 (PMC5366351; doi:10.3389/fpsyg.2017.00444)
Supplement: Supplementary file 1 [file Data_Sheet_1.docx]

# Supplementary Material

|  | Question |
| --- | --- |
| 1 | Please confirm that you are an educator in Higher Education (e.g. Tutor, Lecturer, Professor etc). (Y/N). |
| 2 | Which of these teaching methods have you used in the last 12 months? Formative Assessment (practise tests)/ Learning Styles (matching teaching to student Learning Styles)/ Microteaching (peer review by educators using recorded teaching)/Peer Teaching (students teaching each other)/ Worked Examples/ None of the above |
| 3 | Rank these teaching methods – which do you think is the most effective (1) to the least effective (5): Formative Assessment/ Learning Styles/ Microteaching/Peer Teaching/ Worked Examples |
| 4 | Have you ever administered a Learning Styles questionnaire to your students, choose which ones: Honey +Mumford/Kolb Learning Styles Questionnaire/ Felder/ VARK/ Other |
| 5 | Rate your agreement with the statement: Individuals learn better when they receive information in their preferred Learning Style (e.g.,auditory, visual, kinaesthetic). (Likert) |
| 6 | Rate your agreement with the statement: I try to organise my teaching to accommodate different student Learning Styles (e.g. visual, kinaesthetic, assimilator/converger). (Likert). |
| 7 | Evidence provided on why Learning styles is a myth. Have you been taught/advised to accommodate student Learning Styles in your teaching? (Y/N). |
| 8 | I am aware of the lack of an evidence base to support the use of Learning Styles (e.g. the work of Howard Pashler, Frank Coffield, Dan Willingham and colleagues). (Likert). |
| 9 | Even though there is no 'evidence base' to support the use of Learning Styles, it is my experience that their use in my teaching benefits student learning (Likert). |
| 10 | Classifying students according to their Learning Style risks pigeonholing them so that they might not pursue interests which seem to conflict with their designated Learning Style (e.g. auditory learners may be put off becoming an architect or photographer, or visual learners may be put off studying music). Likert). |
| 11 | The use of non-evidence based methods such as Learning Styles will detract and divert resources from other (effective) areas of education and thus may impair student achievement. (Likert). |
| 12 | The continued promotion of Learning Styles as a product is exploiting students and their teachers, for the financial gain of those companies which sell access to, and training in, the various Learning Style questionnaires. (Likert) |
| 13 | In my experience, students believe, rightly or wrongly, that they have a particular Learning Style. (Likert) |
| 14 | The theory of Learning Styles is conceptually flawed - it does not account for the complexity of ‘understanding’. It is not possible to teach complex concepts such as mathematics or languages by presenting them in only one style.  In addition, some information cannot be presented in a single style (e.g., teaching medical students to recognise heart sounds would be impossible using visual methods, whereas teaching them to recognise different skin rashes would be impossible using sounds). (Likert). |
| 15 | Due to the conceptual problems outlined in the previous question, it is practically very difficult to try and apply any theory of Learning Styles. However, as teachers we are often expected to do so. This disconnect between flawed theory and practice will create anxieties for teachers and unrealistic expectations in students who have completed a Learning Styles questionnaire. (Likert). |
| 16 | Continued use of a technique that demonstrably does not work (i.e. Learning Styles) will undermine the credibility of education as an academic discipline. (Likert). |
| 17 | Rank the aforementioned factors in terms of how compelling they are as reasons not to use Learning Styles (1, most compelling, 6, least compelling). Only rank those factors which you agree with. Creates unrealistic expectations - by students, of teachers/Pigeonhole learners/Primary motive of the Learning Styles industry is profit, rather than helping students/'Understanding’ a concept is complex and cannot be accounted for using current 'Learning Styles'/Undermine credibility of education as an academic discipline/Waste resources that could be better spent elsewhere. |
| 18 | Completing this questionnaire has helped me understand the lack of any evidence base to support the use of Learning Styles. (Likert). |
| 19 | In light of the information presented, rate your agreement with the following statement – ‘I plan to try and account for individual student Learning Styles in my teaching’ (you will be able to add any comments at the end). (Likert). |
| 20 | Demographics: Subject discipline/Approximately how many years have you been teaching in Higher Education/ Title (e.g. Lecturer, Senior Lecturer, Reader, Professor)/Rate your agreement with the following statement “my educational practice is informed by the education research literature”, with 1 being 'Agree' and 5 being 'Disagree'/Do you have a formal teaching qualification (e.g. PGCTHE, master’s in education). |
